# The impact of alpha lipoic acid on developmental competence of mouse vitrified pre-antral follicles in comparison to those isolated from vitrified ovaries

**Published:** 2014-01

**Authors:** Sahar Hatami, Saeed Zavareh, Mojdeh Salehnia, Taghi Lashkarbolouki, Mohammad Taghi Ghorbanian, Isaac Karimi

**Affiliations:** 1 *School of Biology, Damghan University, Damghan, Iran.*; 2 *Institute of Biological Sciences, Damghan University, Damghan, Iran.*; 3 *Department of Anatomy, Tarbiat Modares University, Tehran, Iran.*; 4 *Laboratory of Molecular and Cellular Biology, Department of Basic Veterinary Sciences, School of Veterinary Medicine, Razi University, Kermanshah, Iran.*

**Keywords:** *Vitrification*, *Ovary*, *Preantral follicles*, *Alpha Lipoic Acid*

## Abstract

**Background:** Cryopreservation of ovarian tissues and pre-antral follicles is a promising prospect for preservation of women fertility.

**Objective: **The aim of this study was to evaluate the in vitro developmental competence of mouse vitrified pre-antral follicles in comparison to isolated pre-antral follicles derived from vitrified ovaries in the presence of alpha lipoic acid (ALA).

**Materials and Methods:** Pre-antral follicles derived from fresh, vitrified-warmed ovarian tissues and vitrified–warmed pre-antral follicles were cultured individually with or without ALA, followed by adding hCG to induce ovulation. The follicle growth, oocyte maturation, and embryo development were assessed.

**Results:** The diameter and development of follicles, oocyte maturation and embryo development rates were significantly higher in ALA supplemented groups compared to the respective ALA-free conditions groups. Aforementioned parameters were significantly higher in vitrified-warmed follicles in comparison to follicles derived from vitrified-warmed ovaries.

**Conclusion:** These findings support a superior performance of pre-antral follicles when vitrified rather than when isolated from vitrified ovaries with regard to increasing the rates of developmental parameters. Moreover, ALA improves the in vitro maturation of pre-antral follicles in vitrified and non-vitrified samples.

This article extracted from M.Sc. thesis. (Sahar Hatami)

## Introduction

In recent years, preservation of female fertility in cancer patients who are facing chemotherapy or radiotherapy has been done in several ways such as cryopreservation of ovarian tissue, oocytes and embryos. Cryopreservation of ovarian tissue rather than cryopreservation of oocytes and embryos has many advantages (1). It will preserve the ovarian functional and structural integrity which in turn leads to the restoration of both steroidogenic and gametogenic functions (2). Furthermore, in order to prevent delay in cancer treatment, ovarian tissue can be collected from a patient regardless of the menstrual cycle at any time (3). 

This method is also appropriate for pre-pubertal girls and patients with premature ovarian failure (3, 4). In recent decades, the conventional slow freezing of ovarian tissue has been performed successfully nevertheless it also associated with some disadvantages (4). Vitrification as an alternative approach is achieved by direct plunging into liquid nitrogen (LN2) without subsequent cryodamage resulting from ice formation (5, 6). High cooling and thawing rate are necessary to reduce adverse effects of crystallization in vitrification. With increasing sample size, the sample is surrounded by vapor and not LN2 so that the cooling rate decreases which in turn leads to dramatic decline of the survival rates of vitrified samples (5). Ovarian follicles can also be isolated from fresh ovarian tissue and then vitrified (7). 

This procedure not only increases cooling rate through reducing sample size, but also reduces the risk of transmission of cancer cells from vitrified ovarian tissue back into recipients and also provides the assessment of follicles quality during culture period after warming (8). Nowadays, several culture systems have been proven to cultivate isolated follicles and used to achieve an alive progeny (7, 9, 10). 

However, cryopreservation seems to trigger biological events not responsible for normal cell function and follicular development (11). Increased production of reactive oxygen species (ROS) is one of the consequences of crypreservation and thawing of semen and ovarian tissue (9, 12, 13). It could be due to a hypoxic condition that leads to excess electron production which in turn results in formation of ROS (12). It has been demonstrated that reduced oxygen environment prompts damaging effects on maturation of squirrel monkey's oocytes (14). In the *in vivo *condition, generation of ROS is equilibrated by enzymatic and no enzymatic antioxidant defense systems of cells (15, 16). 

However, the lack of this physiological defense system in the *in vitro* condition under high pressure of oxygen can lead to oxidative stress (17, 18). Alpha-lipoic acid (ALA), as a coenzyme of mitochondrial multienzyme complexes, is well known for its antioxidant properties which acts directly on scavenging of ROS, and indirectly on intracellular recycling of other antioxidants (19, 20). It has been revealed that ALA improves the *in vitro *development of follicles via decreasing ROS concentration and increasing total antioxidant capacity (TAC) level of follicles throughout culture period (21). 

Furthermore, it has been shown that ALA supplemented maturation medium improves developmental competence and modifies oxidative status of oocytes through inhibiting the expression of apoptotic activators and accelerate synthesize glutathione (GSH), glutathione peroxidase 4 (GPx4) and superoxide dismutase (SOD) (22). To date there is no study indicating the effects of ALA on the developmental competence of vitrified pre-antral follicle and isolated pre-antral follicles derived from vitrified ovaries. Therefore, the objective of the present study was to compare follicle growth and development, oocyte maturation and embryo development rates of mouse vitrified-warmed pre-antral follicles and isolated pre-antral follicles derived from whole vitrified-warmed ovaries during *in vitro* culture in the presence of ALA.

## Materials and methods

**Reagents**

All reagents were purchased from Sigma-Aldrich, UK unless otherwise stated and all media were made with Mili-Q water.

**Animals**

In this experimental animal study, female National Medical Research Institute (NMRI) mice were purchased from Pasteur Institute, Iran. Animals were cared for and used according to the Animal Ethics Committee. The mice were housed and bred in the Animal House of Damghan University under light and temperature controlled conditions (12 h of light and 12 h of darkness; and 24^o^C) and they provided with food and water *ad libitum*. 

For all experiments, mice aged 14-15 days old were killed by cervical dislocation and their ovaries were placed in α-minimal essential medium (α-MEM; Gibco, UK) supplemented with 10% fetal bovine serum (FBS; Gibco, UK), 2.2 g/l sodium bicarbonate, 25 mM HEPES, 100 IU/ml penicillin and 75 µg/ml streptomycin. The ovaries were randomly divided into 3 groups: 1) those that not vitrified (control), 2) those that vitrified and 3) those which their follicles were isolated and then vitrified. Isolated pre-antral follicles of each group were cultured in the presence or absence of 100 µM of ALA. 

**Pre-antral follicles isolation**

The preantral follicles of fresh and vitrified-warmed ovaries were isolated mechanically by using a 29 gauge needle under a stereomicroscope (Nikon, Japan) at 10X magnification. Isolated follicles were selected according to the following criteria: rounded follicular structure with 140-150 µm diameters, containing visible centrally located healthy oocyte surrounded with intact several layers of granulosa cells, an intact basement membrane, at least one layer of theca cells and no antral cavity. During the operating procedures the culture medium was always kept at 37^o^C.

**Vitrification and warming of ovarian tissue and pre-antral follicles**

The vitrification procedure was based on a method described previously with some modifications. Exposure time to the equilibrium solution (ES) and vitrification solution (VS) for ovarian tissue and pre-antral follicles were also based on the methods described previously (23, 24).

In brief, ovarian tissues and isolated pre-antral follicles were initially incubated for 10 and 5 minutes respectively in ES containing 7.5% (v/v) ethylene glycol (EG) and 7.5% (v/v) dimethylsulfoxide (DMSO) in Dulbecco phosphate-buffered saline medium (DPBS, Gibco, UK) with 20% fetal bovine serum (FBS), then incubated in vs. (15% EG, 15% DMSO and 0.5M sucrose in DPBS+20% FBS) for another 2 min and 30 sec for ovarian tissue and isolated pre-antral follicles, respectively. After dehydration, the ovarian tissues and pre-antral follicles were immediately drawn into a Pasteur pipette with minimum volume of VS (<0.1µl) and were individually placed on top of a polypropylene strip of cryotop carrier (Kitazato, Japan) and immediately immersed into LN2. 

The thin strip of cryotop was covered with cap and stored in LN2 tank for at least 1 week. All equilibration and vitrification steps were carried out at room temperature. For warming, the cryotop cap was removed while it was still immersed in LN2, then the strip directly submerged in DPBS medium containing 1M sucrose. The pre-antral follicles and ovarian tissue were left in the warming solution (1M sucrose in DPBS) for 30 sec and 5 min respectively and transferred into droplets of DPBS medium containing 0.5 and 0.25M sucrose at an interval of 5 min and 3 min respectively at room temperature. After this, ovarian tissue and pre-antral follicles were pipetted into fresh α-MEM medium supplemented with 10% FBS at 37^o^C for another 10 min before placing in culture medium.

**In vitro culture of isolated pre-antral follicles**

*In vitro* culture of the isolated fresh and vitrified/warmed pre-antral follicles was performed according to our previous methodology (21). Briefly, fresh and vitrified/warmed pre-antral follicles were cultured individually in 20 µl droplets of α-MEM (pH= 7.2) supplemented with 0.23 mM sodium pyruvate, 5% FBS, 100 mIU/ml recombinant follicle stimulating hormone (rFSH; Gonal-f Switzerland), 10 mg/mL insulin, 5.5 mg/mL transferrin and 0.67 mg/ml selenium (ITS; Gibco, UK), 20 ng/ml murine recombinant epidermal growth factor, 100 IU/ml penicillin, 75 µg/ml streptomycin, 2.2g/l sodium bicarbonate and 100 µM ALA according to experimental design under mineral oil in a humidified atmosphere of 5% CO_2_ in air at 37^o^C in Falcon Petri dishes (60×15 mm) for up to 12 days. At 48h intervals, 10 µl of culture medium from each drop was replenished by fresh medium.

**Evaluation of morphological and developmental parameters of cultured pre-antral follicles**

The survival rate of the pre-antral follicles was assessed microscopically based on the morphology of the pre-antral follicle every other day under inverted microscope during culturing period and they were compared at the end of the study as described previously (7). Briefly, a follicle was considered normal when it possessed a centrally located spherical and homogeneous oocyte surrounded by complete and compact layers of granulosa cells with no noticeable damage to the basement membrane. Follicles with partially or completely naked oocytes or those that showed morphological signs of degeneration, such as darkness of oocytes and surrounding cumulus cells or those with misshapen oocytes, were graded as degenerated. 

Antral-like cavity formation was considered as visible lucent area in the granulosa cell mass around the oocyte. Follicles diameter was measured only in healthy follicles on day 2 and 4 of culturing period, mean follicle diameter was assessed by measuring and averaging two perpendicular cross sectional diameters of each follicle with pre-calibrated ocular micrometer under inverted microscope (Nikon, Tokyo, Japan; 100× magnification) (21). 

**In vitro ovulation induction**

In the twelfth day of culture, ovulation and final oocyte maturation were induced by substitution of culture media with fresh maturation medium supplemented with 1.5 IU/ml human chronic gonadotropin (hCG; IBSA, Switzerland). 24 h after hCG addition in all groups, released oocytes were classified as germinal vesicle (GV), germinal vesicle breakdown (GVBD) when the GV was absent, and metaphase II oocytes (MII) when the first polar body was extruded. 

**In vitro fertilization**

The fertile NMRI male mice were sacrificed by cervical dislocation and their caudae epididymides dissected and placed into a 500-µl drop of modified Tyrods medium (mT6) supplemented with 5% bovine serum albumin (BSA) under mineral oil. The caudae epididymides were ripped with the aid of 28 gauge needle and the spermatozoa squeezed out gently by using forceps and incubated for 90 min at 37^o^C in 5% CO_2_ to allow the spermatozoa to swim out and capacitate. Capacitated sperm suspension was added to MII oocyte to give the final motile sperm concentration of 1-2×10^6^ /ml. 

MII oocytes and spermatozoa were co-incubated in modified KSOM-AA medium at 37^o^C under 5% CO_2_ for 6h. Oocytes presenting two pronuclei (2PN) were considered normally fertilized. Fertilized oocytes were washed and gently pipetted to remove spermatozoa and attached cumulus cells. Zygotes were then transferred to drops of fresh KSOM-AA medium supplemented and cultured at 37^o^C under 5% CO_2_ with maximum humidity until day 5. Day of insemination was considered as day 0. 

Embryos were observed on the heated stage (37^o^C) of an inverted microscope (Nikon, TE2000-U with Hoffman modulation contrast). The numbers of zygotes that reached to two cells, morula and blastocytes were counted.

**Statistical analysis**

Each experiment was carried out at least four replicates. The data are presented as the mean±SD. Statistical analysis was performed using SPSS-ver.16 software package (SPSS Inc., Chicago, IL, USA). All proportional data were analyzed after arcsine of sqtr transformation. Differences in groups were evaluated by one-way analysis of variance (ANOVA) and Tukey's HSD was used as *post hoc* test. Differences were considered significant at a level of p<0.05. 

## Results

The morphological and maturational parameters of the cultured vitrified and non-vitrified isolated pre-antral follicles with or without ALA are summarized in table I and II. By day 12 of culture, with respect to the follicle diameter and rates of survival, antrum formation and oocyte maturation, there were significantly differences in both vitrified and non-vitrified ALA supplemented samples compared with ALA-free conditions groups (Table I & II(. After the fourth day of culture, granulosa cells proliferated and grew through basal membrane to form the irregular and diffuse appearance that was impossible to measure follicle diameter. The mean follicle diameter at the beginning of the culture (147.9±5.9 µm) was not significantly different among all groups (p=0.83). During the culture period, the follicle diameter increased progressively. 

As show in table I, the diameter of fresh pre-antral follicles on day 2 and 4 (223.9±17.1 and 323.3±11.6 µm respectively) of cultivation period in the presence of supplementation with ALA was significantly higher than the other groups. Also, the mean diameter of the vitrified-warmed pre-antral follicles on day 2 and 4 in the presence of ALA (180.5±7.2 and 272.1±18.4 µm respectively) were significantly higher than those which were isolated from vitrified-warmed ovaries. There were no significant differences between diameters of vitrified-warmed follicles and those which were derived from vitrified-warmed ovaries that were cultured in the presence of ALA (Table I).

The survival (94.3%) and antrum formation (92.3%) rates were significantly higher in fresh pre-antral follicles supplemented with ALA than the other groups (Table II). The rates of survival (68.3%) and antrum formation (62.8%) in the vitrified-warmed pre-antral follicles were significantly higher than those which were isolated from vitrified-warmed ovaries (57.3% and 46.0%, respectively). There was no significant difference between these rates of vitrified-warmed pre-antral follicles and pre-antral follicles derived from vitrified-warmed ovaries which were cultured in the presence of ALA (66.7% and 60.6%, respectively). 

Table II shows the percentage of GV, MI and MII oocytes from the follicles after addition of the hCG induction on day 12. Higher percentages of MII oocytes were also obtained in vitrified-warmed pre-antral follicles than those which were isolated from vitrified-warmed ovaries (28.3% and 15.0% respectively, p=0.001), although both were statistically lower than control group (42.9%). The percentage of MII oocytes was similar between cultured isolated pre-antral follicles derived from vitrified-warmed ovaries (26.7%) with ALA and those of vitrified-warmed pre-antral follicles (28.3%); however, both rates were significantly lower in comparison to control group. 

Nevertheless, the differences were all significant compared to that of pre-antral follicles which were cultured in the presence of ALA (53.9%). Fertilization and embryo developmental rates of MII oocytes derived from cultured isolated follicles in non-vitrified and vitrified groups in the presence of ALA are shown in table III. There were significant differences in the fertilization rate and blastocyst formation rate in between the respective ALA-free and ALA supplemented groups both in vitrified and non-vitrified groups. 

Significant higher rates of fertilization and blastocyte were observed in vitrified-warmed pre-antral follicles group in comparison with vitrified-warmed ovaries groups; however both were statistically lower than control group. There were no significant differences in the rates of fertilization and blastocyst formation between vitrified-warmed pre-antral follicles samples and pre-antral follicles derived from vitrified-warmed ovaries which were cultured in the presence of ALA. Also, these situations were observed between cultured vitrified-warmed pre-antral follicles samples in the presence of ALA and ALA-free fresh pre-antral follicles samples.

**Table I:** Diameter of cultured preantral follicles derived from fresh ovaries, vitrified ovaries and vitrified follicles in the presence or absence of alpha lipoic acid (ALA)

**Groups**	**ALA**	**NO. of follicles**	**Follicle diameter (** **µm ± SD)**		
			**0** ^th^ ** day**	**2** ^th^ ** day**	**4** ^th^ ** day**
Fresh ovaries					
	-	240	148.2 ± 5.3	189.6 ± 11.4^a^	290.5 ± 26.3^a^
	+	180	147.5 ± 6.6	223.9 ±17.1^b^	323.3 ± 11.6^b^
Vitrified ovaries					
	-	180	147.6 ± 5.2	170.5 ± 13.1^d^	255.3 ± 37.3^d^
	+	180	147.3 ± 6.5	176.5 ± 4.8^c^	262.9 ± 4.7^c^
Vitrified pre-antral follicles					
	-	180	148.3 ± 5.0	175.3 ± 7.2^c^	264.2 ± 6.7^c^
	+	180	148.6 ± 5.7	180.5 ± 7.2^a^	272.1± 18.4^a^

In all cases at least 4 experimental replicates were performed. Different superscripts in the same column reflect different levels of significant difference (p<0.05)

**Table II:** Maturation rates of cultured pre-antral follicles derived from fresh ovaries, vitrified ovaries and vitrified follicles in the presence or absence of alpha lipoic acid (ALA)

**Groups**	**ALA**	**N**	**Survived**	**Degenerated**	**Antrum formation**	**Maturation stages of oocyte**		
						**GV**	**MI**	**MII**
Fresh ovaries								
	**-**	240	204^a ^(85.0±1.9)	36^a ^(15.0±1.9)	184^a ^(76.7±5.6)	30 (12.5±3.5)	51 (21.2±3.7)	103^a ^(42.9±2.8)
	**+**	180	169 ^b ^(94.3±1.2)	11^b ^(5.7±1.2)	166^b ^(92.3±4.0)	16 (9.5±2.4)	34 (18.9±5.1)	97^b ^(53.9±4.2)
Vitrified ovaries								
	**-**	180	102^c ^ (57.3±3.5)	72^c ^(43.3±2.9)	83^c ^(46.0±4.2)	27 (15.0±1.7)	22 (12.2±3.5)	27^c ^(15.0±1.7)
	**+**	180	120^d ^ (66.7±3.3)	60^d ^(33.3±3.3)	111^d ^(60.6±2.5)	20 (11.1±2.5)	31 (20.6±4.2)	48^d ^(26.7±1.7)
Vitrified pre-antral follicle								
	**-**	180	123 ^d ^(68.3±6.0)	57^d ^(31.7±6.0)	113^d ^(62.8±4.2)	21 (11.7±1.7)	20 (11.1±2.5)	54^d ^(28.3±1.7)
	**+**	180	150^a^ (83.3±3.3)	30^a ^(16.7±3.3)	134^a ^(74.4±4.2)	20 (11.1±2.5)	21 (11.7±1.7)	66^a ^(36.7±3.3)

Data were presented as number (% ± SD) in all columns.

In all cases at least 4 experimental replicates were performed.

Different superscripts in the same column reflect different levels of significant difference (p<0.05)

GV: germinal vesicle stage oocyte; MI: metaphase I oocyte; MII: metaphase II oocyte.

**Table III:** Development rates of derived embryos from cultured pre-antral follicles in the presence or absence of alpha lipoic acid (ALA)

**Groups**	**ALA**	**MII Oocytes** **(n)**	**Fertlized**	**Two cells**	**Morulla**	**Blastocytes**
Fresh ovaries						
	**-**	103	67^a ^(65.1±2.9)	45^a ^(43.6±2.1)	31^a ^(30.1±3.1)	25^a ^(24.3±0.9)
	**+**	97	81^b ^(83.6±3.8)	54^b ^(55.6±2.2)	47^b ^(48.6±2.5)	41^b ^(42.3±1.5)
Itrified ovaries						
	**-**	27	10^c ^(36.9±3.4)	7^c ^(25.7±4.0)	2^c ^(7.0±6.1)	2^c ^(7.0±6.1)
	**+**	48	26^d ^(54.2±1.9)	16^d ^(33.3±2.0)	10^d ^(20.8±2.4)	7^d ^(14.5±2.7)
Vitrified pre-antral follicle						
	**-**	54	29^d ^(53.6±4.0)	19^d ^(35.1±1.8)	11^d ^(20.3±2.4)	9^d ^(16.7±0.9)
	**+**	66	42^a ^(63.6±4.2)	28^a ^(42.4±2.8)	22^a ^(33.2±3.2)	17^a ^(25.8±1.3)

Data were presented as number (% ± SD) in all columns.

In all cases at least 4 experimental replicates were performed.

Different superscripts in the same column reflect different levels of significant difference (p<0.05)

MII: metaphase II oocyte.

## Discussion

In the present study, vitrification of isolated mouse pre-antral follicle was more effective in comparison with vitrification of whole ovarian tissue due to improving follicular survival, antrum formation and oocyte maturation rates. This difference in the cryobiological features of vitrified-warmed pre-antral follicles and pre-antral follicles derived from vitrified-warmed ovarian tissue may be attributed to their morphological differences. In this regard, it seems that vitrification of ovarian tissue compared to vitrification of pre-antral follicles is facing to some obstacles such as the high volume ratio to low surface area of a whole ovary, which will slow down the cooling and warming rates (25). 

This feature of ovarian tissue causes temperature transition from the central region of the ovary to be slowly driven, which in turn will lead to increased possibility of ice crystal formation thus be at a greater risk of severe damage (3, 26). Furthermore, ovarian tissue thickness prevents permeation of cryoprotectant to the entire tissue within a given exposure time to equilibration and vitrification solution thus resulting in ice formation (25). In order to overcome this problem, the exposure time to equilibration and vitrification solution should be increased which in turn can lead to toxicity in surface areas (2). 

Thus, it appears that differences between maturational competence of vitrified-warmed pre-antral follicles and pre-antral follicles obtained from vitrified-warmed ovarian tissue are due to the small size of the pre-antral follicles and increased surface to volume ratio, which in turn will overcome obstacles mentioned above. Effect of cryopreservation on ultra-structural properties of vitrified ovaries has been investigated previously (27-29). In this regard, ultra-structural evaluation by transmission electron microscopy showed that the low developmental competence of oocyte after vitrification and warming is partially due to diffused and fragmented mitochondria (29). Also, according to the ultra-structure investigations, it was shown that oocytes of follicles derived from vitrified-warmed ovarian tissues reveales increased numbers of swollen mitochondria (27, 28). 

Our explanation for different developmental competence among vitrified groups and fresh samples is that, mitochondrial disorders that are occurred throughout cryopreservation could increase ROS generation in vitrified samples and it seems that these disorders were minor in vitrified-warmed pre-antral follicles than pre-antral follicles derived from vitrified-warmed ovary, because development rate was higher in vitrified-warmed pre-antral follicles than those of pre-antral follicles dived from vitrified-warmed ovaries. Several defense mechanisms against ROS are present including non-enzymatic antioxidants and enzymatic defense mechanisms within cells (30). 

We reported useful impact of ALA as a potent antioxidant on the developmental competence of isolated pre-antral follicles in fresh samples (21). However, this is the first study to show the effects of ALA on *in vitro* maturation of vitrified-warmed pre-antral follicles and pre-antral follicles derived from vitrified-warmed ovaries. The present study showed that ALA could improve the rates of survival, antrum formation and MII oocytes of vitrified-warmed and fresh pre-antral follicles after long-term *in vitro* culture. 

Several hypotheses can be considered for the role of ALA on improving the development of vitrified-warmed and fresh pre-antral follicles. Some investigators attributed the efficiency of ALA to unique antioxidant properties of lipoate/ dihydrolipoate system, i.e. ROS scavenging ability (21, 22, 31). This feature of ALA has been attributed partially to suppress TNF-alpha-induced ROS generation and 6-hydroxydopamine induced ROS generation (32). Also it has been known that ALA acts as a coenzyme of pyruvate dehydrogenase complex that catalyses the consumption of pyruvate in mouse follicles and oocytes (35). 

To test this hypothesis, further studies must be designed to determine monergic coenzymatic role or possible synergic coenzymatic roles of ALA combined with the rest of coenzymes of pyruvate dehydrogenase complex like thiamine pyrophosphate, FAD, NAD^+^, and coenzyme A. In this sense, sodium pyruvate routinely is a constant component of culture media like KSOM-AA medium (see Materials and Methods). ALA may be involved in improving development of cultured pre-antral follicles via metal chelation, antioxidant recycling, and gene expression (19). The other explanation for effects of ALA in improving IVM conditions may be related to inhibition of cell death (36). Also, it has been shown that ALA increases the catalase (CAT) activity and glutathione peroxidase (GPx), activity (37). 

CAT and GPx are two fundamental enzymes can be used as a biomarker of oxidative stress (38). Also it has been suggested that activation of GPx in follicular fluid were clearly associated with developmental competence of oocyte (39). In sum, the results of present study demonstrated that vitrification of isolated pre-antral follicles is more effective method to increase follicular survival, antrum formation and oocyte development rates rather than vitrification of whole ovarian tissue. In addition to, this study showed that culture medium supplementation with ALA improves development of cultured pre-antral follicles derived from fresh and vitrified samples. 

Future mechanistic studies on the identification of metabolic role of ALA involved in follicular maturation beside its antioxidative activity in assisted reproduction techniques should therefore be conducted.
